# *Reprimo*, a Potential p53-Dependent Tumor Suppressor Gene, Is Frequently Hypermethylated in Estrogen Receptor α-Positive Breast Cancer

**DOI:** 10.3390/ijms18081525

**Published:** 2017-08-15

**Authors:** Kurt Buchegger, Ismael Riquelme, Tamara Viscarra, Carmen Ili, Priscilla Brebi, Tim Hui-Ming Huang, Juan Carlos Roa

**Affiliations:** 1 Department of Pathology, Molecular Pathology Laboratory, School of Medicine, Universidad de La Frontera, Avenida Alemania 0458, 4810296 Temuco, Chile; kurt.buchegger@ufrontera.cl (K.B.); ismael.riquelme@ufrontera.cl (I.R.); t.viscarra01@ufrontera.cl (T.V.); carmen.ili@ufrontera.cl (C.I.); priscilla.brebi@ufrontera.cl (P.B.); 2 Centro de Excelencia en Medicina Traslacional-Scientific and Technological Bioresource Nucleus (CEMT-BIOREN), Universidad de La Frontera, Avenida Alemania 0458, 4810296 Temuco, Chile; 3 Department of Molecular Medicine/Institute of Biotechnology, University of Texas Health Science Center at San Antonio, San Antonio, TX 78229, USA; huangt3@uthscsa.edu; 4 Department of Pathology, UC Centre for Investigational Oncology (CITO), Advanced Centre for Chronic Diseases (ACCDis), The Millennium Institute on Immunology and Immunotherapy, Pontificia Universidad Católica de Chile, 8330024 Santiago, Chile

**Keywords:** estrogen, estrogen receptor α, DNA methylation, *Reprimo*, breast cancer

## Abstract

Aberrant DNA methylation is a hallmark of many cancers. Currently, there are four intrinsic molecular subtypes in breast cancer (BC): Luminal A, B, Her2-positive, and triple negative (TNBC). Recently, The Cancer Genome Atlas (TCGA) project has revealed that Luminal subtypes have higher levels of genome-wide methylation that may be a result of Estrogen/Estrogen receptor α (E2/ERα) signaling pathway activation. In this study, we analyze promoter CpG-island (CGIs) of the *Reprimo (RPRM)* gene in breast cancers (*n* = 77), cell lines (*n* = 38), and normal breast tissue (*n* = 10) using a MBDCap-seq database. Then, a validation cohort (*n* = 26) was used to confirm the results found in the MBDCap-seq platform. A differential methylation pattern was found between BC and cell lines compared to normal breast tissue. In BC, a higher DNA methylation was observed in tissues that were ERα-positive than in ERα-negative ones; more precisely, subtypes Luminal A compared to TNBC. Also, significant reverse correlation was observed between DNA methylation and *RPRM* mRNA expression in BC. Our data suggest that ERα expression in BC may affect the DNA methylation of CGIs in the *RPRM* gene. This approach suggests that DNA methylation status in CGIs of some tumor suppressor genes could be driven by E2 availability, subsequently inducing the activation of the ERα pathway.

## 1. Introduction

Breast cancer (BC) is the second most common cancer in the world, and by far the most frequent cancer among women, with about 1.67 million new cases diagnosed in 2012 (25% of all cancers), affecting mainly women from developed countries in Western Europe and North America [1]. BC is classified into four intrinsic molecular subtypes (Luminal A and B, Her2-enriched, and Basal-like) according to gene expression patterns [2]. The use of molecular markers, such as the receptor status of estrogen (ERα), progesterone (PR), epidermal growth factor 2 (Her2/*neu*), and proliferation marker Ki67, can be helpful to subclassify BC cases into: Luminal A (ERα-positive, PR-positive, Her2/*neu*-negative, and Ki67-low), Luminal B (ERα-positive, PR-positive, Her2/*neu*-positive or negative, and Ki67-high), Her2/*neu*-positive (ERα-negative, PR-negative, and Her2/*neu*-positive), and triple negative (TNBC) (negative for ERα, PR, and Her2/*neu*). This classification is important to evaluate clinical prognosis, provide the best treatment available, and assess patient outcomes due to notorious survival differences among these subtypes [2,3,4]; however, this stratification does not always coincide with the intrinsic molecular subtype determined by high-throughput platforms.

The most common subtypes in BC are Luminal A and B (ERα-positive) with a frequency of ~70% and ~12% of cases, respectively [5]. Fortunately, these two subtypes have a good prognosis, because they are frequently differentiated and low-graded tumors [6], and have a good response to endocrine therapy based on ERα-antagonist drugs (e.g., Tamoxifen), inhibitors of estrogen (E2) synthesis (e.g., aromatase inhibitors), and selective ERα downregulators (e.g., Fulvestrant) [7].

ERα is a nuclear protein that functions as a transcription factor and as an important regulator of growth, differentiation, and metabolism. The canonical model for ERα activation starts with the binding of E2 to ERα in the cell cytoplasm and subsequent migration of this E2/ERα complex into the nucleus for binding directly to estrogen response elements (EREs), which are palindromic consensus sequences (GGTCAnnnTGACC) present in the DNA [8,9]. These ERE sequences have been found in several genes, including tumor suppressor genes [10]. For instance, Malik et al. [11] provided new evidence about the role of ERE sequences in the regulation of a E2-dependent gene, *Reprimo* (*RPRM*), whose activation of the E2/ERα complex induces the recruitment of other molecules, such as FoxA1 and HDAC7, causing a transcriptional silencing in a model of a BC cell line.

*RPRM* is a potential p53-dependent tumor suppressor gene [12], constituted by a unique exon of 327 bp located at 2q23.3, which encodes a protein of 109 amino acids. In normal cells, RPRM protein is involved in the G2/M arrest of a cell cycle when DNA is damaged. The *RPRM* gene has been found frequently hypermethylated in several human cancers [13,14,15,16,17,18,19]; however, no evidence of *RPRM* methylation has been reported in BC.

In this regard, previous studies have linked the chromatin organization changes to an ERα-positive status in patients, suggesting that the activation of signaling through ERα may lead to an E2-mediated epigenetic repression affecting the genome organization and regulation of certain genes in cancer [20,21,22,23]. In 2010, Li et al. identified two hypermethylated genes between ERα-positive and ERα-negative breast tumors using genome-wide DNA methylation profiling [24]. In addition, Shi et al. [25] showed that the activation of an E2/ERα complex regulates positively the expression of various DNA methyltransferases (DNMTs), contributing to a tumoral phenotype in BC that is E2-dependent. Other reports have shown that E2 signaling induces the transient formation of multiple DNA loops in the 16p11.2 region [26]. Also, an E2-mediated long-range epigenetic repression (LRES) process has been shown to induce the recruitment of H3K27me3, which results in a chromatin compaction that is frequently complemented with DNA methylation in order to repress transcriptionally the expression of certain genes [27]. Furthermore, Jadhav et al. [28] exposed an E2-mediated epigenetic repression phenomenon in large gene clusters that could be used as potential prognosis markers in breast cancer.

In the present study, the DNA methylation pattern of promoter CpG-island (CGIs) of *RPRM* was explored, using a high-throughput sequencing technology based on capture of Methyl-CpG binding domain (MBDCap-seq) [29] performed in a cohort of 87 breast samples (77 breast cancer and 10 normal breast tissue), as well as a panel of 38 breast cancer cell lines. The aim of this study was to evaluate the methylation pattern of *RPRM* in BC and its association with clinicopathological features and hormonal receptor status by using a MBDCap-seq database.

## 2. Results

### 2.1. Hypermethylation in CpG-Island (CGIs) of Reprimo (RPRM) Is Frequently Found in Estrogen Receptor α–Positive (ERα) Breast Cancer

**Figure 1:**
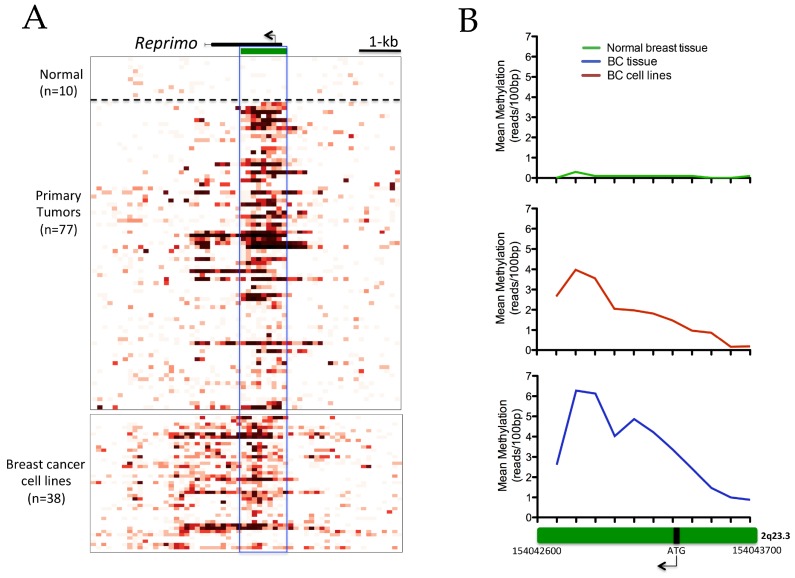

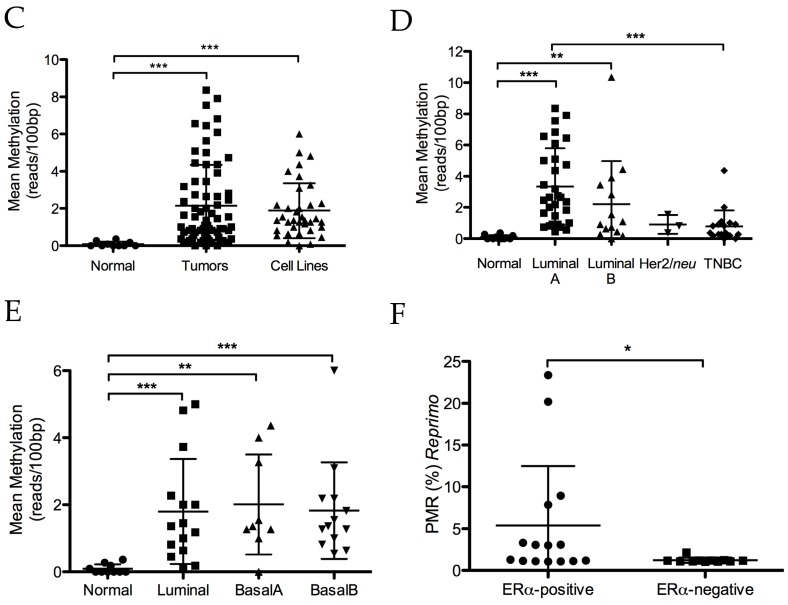
DNA methylation of promoter CpG-island (CGIs) region in breast cancer. Methyl-CpG binding domain (MBD-seq) was used to generate DNA methylation profiles of normal breast tissue (*n* = 10), primary tumors (*n* = 77), and cell lines (*n* = 38). (**A**) The figure represents methylation intensity by 100-bp resolution. The pre-calculated methylation intensity is shown as a red gradient heatmap. At the top part, in black is shown the gene body with an arrow that indicates the ATG sequence. In green, the CGIs of the gene is shown. The solid line highlights the region analyzed (1.1 kb; chr2: 154042600–154043700); (**B**) the Green box represent CGIs amplified from Figure 1A, where we observe a higher mean methylation of CGIs for each 100-bp resolution (calculated as the mean methylation for each 100-bp) in primary tumors and cell lines respect to normal breast tissue; (**C**) methylation intensity (calculated as the mean methylation intensity of CGIs—1.1 kb—for each case) was significantly higher in primary tumors and cell lines compared to normal breast tissue (*p* < 0.0001); (**D**) a scatter plot among different molecular subtypes in primary tumors showed significant differences in the average value of methylation in the *Reprimo* (*RPRM*) CGIs region for Luminal A compared to normal breast tissue and also for triple negative breast cancer (TNBC); (**E**) a scatter plot among different molecular subtypes in breast cancer (BC) cell lines showed significant differences compared to normal breast tissue, but not among them; (**F**) scatter plot of the validation cohort (26 breast cancer samples) showing the significant differences in percentage-methylated relative (PMR) of RPRM CGIs between breast cancer estrogen receptor α (ERα)-positive and ERα-negative. TNBC, triple negative breast cancer. * *p* < 0.05; ** *p* < 0.001; *** *p* < 0.0001.

The datasets of the normal breast tissue, BC tissues, and cancer cell lines were downloaded from the Cancer Methylome System (CMS) website (http://cbbiweb.uthscsa.edu/KMethylomes/) conducted by MDBCap-seq. A detailed visualization analysis along the *RPRM* gene revealed a hypermethylation pattern in the CGIs region of this gene in primary breast tumors and breast cancer cell lines compared to normal breast tissue (Figure 1A). The analysis performed for each 100-bp of CGIs (Start-End: chr2: 154042600–154043700) in BC tissues and BC cell lines showed an increase of methylation patterns in both the upstream zone from the ATG sequence (chr2: 154043300–154043500) and the downstream zone from the exonic region (chr2: 154042800–154042900) (Figure 1B). For additional information, the data for each 100-bp of resolution analyzed in the CGIs are detailed in Table S1.

The mean of methylation intensity calculated for the CGIs region in both primary tumors and cell lines was significantly higher than in the normal breast tissue (*p* < 0.0001; Figure 1C). However, the differences in methylation between BC tissues and cell lines were not significant (Figure 1C), which supports the above-mentioned results and suggests that an increase in methylation intensity of *RPRM* CGIs could be associated with a tumor phenotype in this malignancy. In fact, the methylation status of *RPRM* CGIs was associated with some clinicopathological features in breast cancer patients, such as age (*p* < 0.05), ERα status (*p* < 0.0001), PR status (*p* < 0.05) and molecular subtypes (*p* < 0.001) (Table 1).

In addition, when BC tissues were classified according to the respective clinical molecular subtypes, a higher level of *RPRM* methylation was observed between Luminal A tumors than in normal breast tissue and other molecular subtypes, particularly TNBC (*p* < 0.0001; Figure 1D). Moreover, all BC cell lines showed significantly higher levels of *RPRM* methylation than the normal breast tissue (*p* < 0.0001) regardless of their molecular subtype, but no significant differences were observed among molecular subtypes of cell lines compared to each other (Figure 1E).

In order to validate the results regarding the methylation of *RPRM* CGIs and ERα-status, another cohort of BC cases different to those used in the CMS study was selected. A group of 26 cases were analyzed by qMSP, of which 15 were ERα-positive and 11 ERα-negative. This qMSP assay was designed to evaluate a specific sequence rich in CpG dinucleotides in the promoter region, which has been well-described and studied by several authors [15,18,19,30]. The results showed a higher methylation of *RPRM* promoter region in ERα-positive than ERα-negative BC cases (*p* = 0.0102; Figure 1F). According to its clinicopathological features, the methylation status of the *RPRM* promoter region was associated significantly with ERα status (*p* < 0.05) (Table S2).

**Figure 2:**
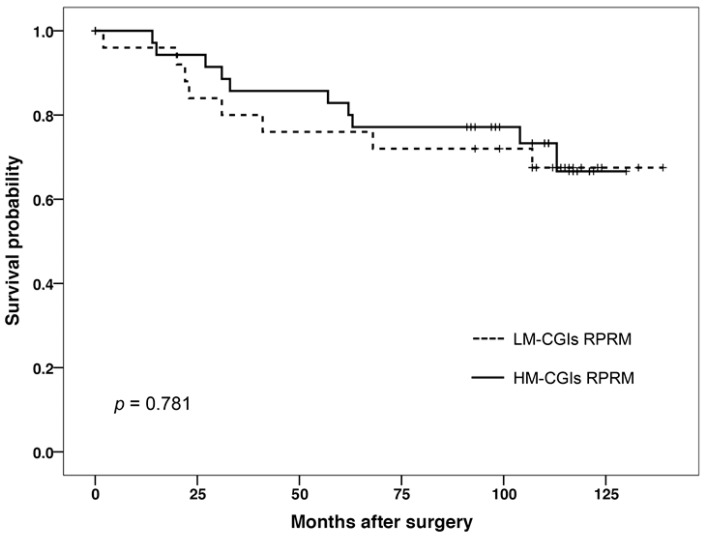
Kaplan–Meier survival curves of 77 breast cancer (BC) patients indicates that differential methylation of *RPRM* CGIs was not associated with overall survival in BC patients. Solid lines indicate patients whose tumors had a high methylation of CGIs, while the dotted line indicates those tumors with low methylation in the CGIs region.

Then, survival analysis was performed in 77 patients; however, no significant differences were found between the survival of patients with high or low methylation (Figure 2). Also, for the survival analysis for ERα-positive and ERα-negative BCs, no statistical significance was observed comparing the survival of both status (*p* > 0.05).

### 2.2. High Methylation Intensity in CGIs Is Inversely Correlated with Transcriptional Expression of RPRM

**Figure 3:**
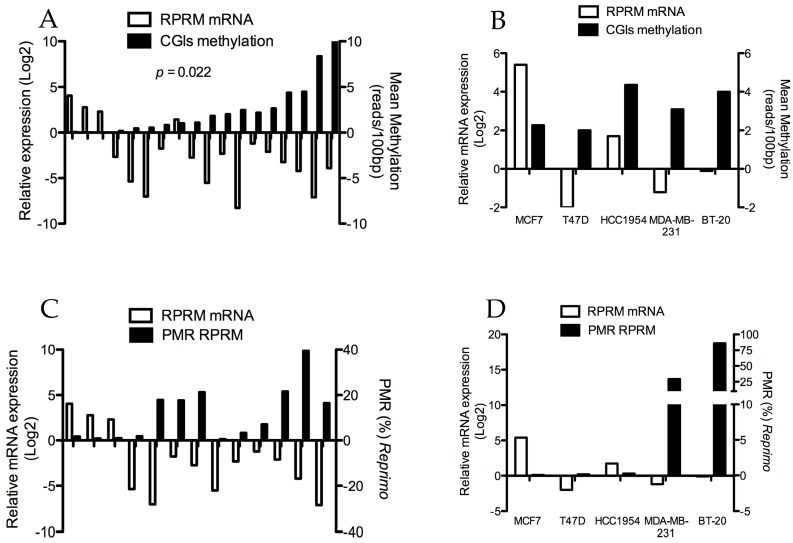
A Spearman correlation analysis between mRNA expression and methylation of *RPRM* CGIs of primary breast tumors and cell lines. (**A**) A significant inverse correlation was observed between mRNA expression and methylation of *RPRM* CGIs in paired clinical samples (*n* = 18) (*p* < 0.05); (**B**) meanwhile, in the BC cell lines, the Spearman correlation was not significant (*p* = *n*.*s*); (**C**) correlation between qMSP and qPCR assay in paired clinical samples (*n* = 13) was not significant (*p* > 0.05); (**D**) in cancer cell lines, an increase of percentage-methylated relative (PMR) RPRM was observed frequently with a downregulation of RPRM mRNA in ERα-negative cells; BT-20 and MDA-MB-231. In contrast, MCF7 shows upregulation of RPRM mRNA without methylation of the RPRM promoter region. However, no significant correlation was observed (*p* > 0.05).

A group of 18 BC tissues available—from the same cohort of the CMS—were studied to determine whether *RPRM* CGIs hypermethylation could effectively affect the mRNA expression of this gene. An inverse correlation was observed between the *RPRM* mRNA expression and the hypermethylation of *RPRM* CGIs (*p* < 0.05; Figure 3A). Interestingly, the *RPRM* mRNA expression decreases drastically even in those samples that show a low methylation intensity (few methylated CpG dinucleotides in the CGIs region). Conversely, when the methylation intensity is null, the *RPRM* mRNA expression increases considerably.

On the other hand, the same experiments were performed in BC cell lines; however, no significant correlation was found between methylation of *RPRM* CGIs and mRNA expression (Figure 3B). Then, in order to determine the correlation between the methylation patterns per each 100 pb-resolution region and the transcriptional expression of *RPRM*, a correlation analysis was performed. Several regions showed a significant inverse correlation with the *RPRM* mRNA expression, including the upstream zone from the ATG sequence (chr2: 154043300–154043599), the exonic region (chr2: 154043000–154043099), and the downstream zone from the exonic region (chr2: 154042600–154042699). This information is detailed in Table S3.

In this regard, given that the methylation intensity of the upstream region from the ATG sequence showed an inverse correlation with the *RPRM* transcriptional expression, a qMSP assay in BC tissues was performed in order to correlate the *RPRM* relative expression and methylation in the promoter region of this same gene. For this assay, DNA was extracted from 13 of 18 BC samples previously studied and 5 BC cell lines. Unfortunately, no significant correlation was observed between mRNA expression and methylation of the *RPRM* promoter region in both BC tissues and cell lines. (Figure 3C,D, respectively).

## 3. Discussion

DNA methylation is an epigenetic mechanism responsible for silencing tumor-associated genes, particularly tumor suppressor genes in cancer [31]. Many of these methylation alterations are tissue-specific or associated with oncogenic processes, but are not cancer-specific [18,32]. In this regard, this study focused on quantitatively analyzing differential methylation patterns in the *RPRM* gene in a cohort of BC patients, identifying for the first time a clear difference between methylation patterns in normal and breast tumor tissues.

*RPRM* is a potential tumor suppressor gene constituted by a unique exon of 327 bp located at 2q23.3, which encodes a glycosylated protein of 109 amino acids frequently found in the cytoplasm. This protein regulates the G2/M transition through the activity of the Cdc2-cyclin B1 complex, inducing cell cycle arrest—in a p53-dependent manner—in the presence of DNA damage [12].

Several reports have demonstrated *RPRM* silencing in human malignancies, mainly due to an aberrant methylation of the *RPRM* promoter region [13,15,17,18,33,34,35,36]. In fact, this phenomenon has been described as a frequent event and cancer-specific feature in cancers such as pancreatic, gastric, and other cancers [13,30,37]. For instance, *RPRM* is frequently methylated in esophageal cancer patients non-responsive to chemotherapy, and is strongly associated with a poor outcome compared to those patients with lower levels of *RPRM* methylation [14]. Furthermore, aberrant *RPRM* methylation in pancreatic cancer is correlated with genetic instability and unfavorable patient outcomes after surgical resection [37]. In lung cancer, *RPRM* methylation was found in 41% of patients in the study; however, similar to our findings, *RPRM* promoter hypermethylation was not significantly correlated with a better or worse 5-year overall survival rate [16]. In gastric cancer, *RPRM* methylation has been widely studied. A study by Bernal et al. proposed *RPRM* methylation as a potential biomarker for the early detection of gastric cancer [15]. In addition, Ooki et al. indicated that clinical assessment of *RPRM* methylation may also serve as a predictive marker for response to chemotherapy consisting of cisplatin and fluoropyrimidines, and as a marker of tumor aggressiveness [18]. Wang et al. [38] recently published an article where they developed a semi-quantitative method based on MS-MCA for detecting DNA methylation of the *RPRM* gene in human plasma or serum samples to help in the diagnosis/prognosis of gastric cancer. Recently, an interesting study by Garcia-Bloj et al. [39] developed a combinatorial strategy for the reactivation of tumor suppressor genes, including *RPRM*, using CRISPR/Cas9 VP64 with synergistic activation mediators, which led to phenotypic reprogramming in AGS gastric cancer cells. Based on the above-mentioned studies, *RPRM* methylation seems to be important in the carcinogenic process in human malignances; however, with the exception of our previous study [40], there have been no reports about methylation status in BC.

Therefore, the methylation patterns in the 1.1 kb region (CGIs) of 77 primary tumors and 10 normal breast tissue (breast reduction) were analyzed, finding a clear hypermethylation of CGIs regions in BC compared to normal tissues. Subsequently, significant differences in methylation intensity were observed between Luminal A compared to TNBC. Differences in methylation status between the Luminal A subtype and the Her2/*neu* subtype were not significant, likely due to the small number of cases in this last group. This is particularly interesting, because The Cancer Genome Atlas (TCGA) data for human breast cancer have shown that luminal tumors—that are ERα-positive—frequently have a hypermethylated phenotype. Conversely, TNBC cases are frequently hypomethylated [41]. For this reason, the methylation status of the CGIs in the *RPRM* promoter region was analyzed by qMSP in a different sample cohort, where the CGIs of *RPRM* were frequently found to be hypermethylated in ERα-positive BC cases compared to ERα-negative BC cases. These results suggest that methylation in the CGIs region of *RPRM* may be associated with ERα status in this malignancy. It is important to highlight that qMSP assay uses primers and a specific fluorescent probe (designed by our group). This pair of primers flanks an upstream region to TSS sequence, and has been frequently used in previous reports conducted by Sato et al. [13], Bernal et al. [15], and Liu et al. [19].

Subsequently, RNA was extracted from 18 samples—previously used in the CMS analysis—in order to correlate the methylation intensity of the RPRM CGIs with transcriptional expression. A significant inverse correlation was observed in the BC tissues, but not in the BC cell lines. Nevertheless, the methylation intensity used in this analysis was obtained from the mean of reads per each 100-bp of RPRM CGIs (1.1 kb). Accordingly, a correlation analysis between mRNA expression and number of reads per each 100-bp region was performed to identify groups of CPIs with a critical regulatory role in transcriptional expression. Several groups of 100-bp regions within the CGIs region were found in the intragenic region (exonic and downstream), but mainly in the promoter region (300 pb approximately), where are located more than 60% of CGIs of the genome [42]. In fact, this region is nearby to the *RPRM* region for which the qMSP assay was designed. In this regard, a qMSP assay was performed to identify a correlation between mRNA expression and methylation in promoter region. Unfortunately, no significant correlations were found, probably because qMSP evaluates a sequence of 120 bp in the promoter region, and within this sequence the probe hybridizes specifically 7 CpG dinucleotides. To solve this issue, a bisulfite sequencing analysis within this region might be suggested for future studies, which would allow them to identify differential methylation patterns with a 1 bp resolution in breast tumors with low and high *RPRM* mRNA expression.

It is well known that hypermethylation in promoter regions affects transcriptional expression, which has an effect on the tumor phenotype. However, high-throughput technologies have revealed that DNA methylation is not only a site-specific epigenetic process, but also acts as a phenomenon spanning long stretches of chromosomal regions, both promoter regions, intragenic, intergenic, non-promoter regions, and even gene clusters that can serve as prognostic markers in cancer [26,28,43,44]. Particularly, the role of intragenic methylation in direct transcriptional repression is unclear, as well as which are the specific sequences that induce this repression, but it seems to depend on the genomic context [45,46].

In BC cell line MCF7, this phenomenon appears to be dependent on E2/ERα pathway activation because of the low methylation intensity found in the CGIs region and confirmed by qMSP assay with an upregulation of *RPRM* mRNA. The lack of E2 hormone in the culture medium may explain this phenomenon. For instance, Malik et al. [11], showed that the activation of ERα resulted in the induction or repression of gene transcription. In fact, they found that *RPRM* is repressed by the tripartite interaction among HDAC7, FoxA1, and ERα. Nevertheless, this study was performed only at a transcriptional level [11]. Other studies in BC cells have demonstrated the effect of E2 in the DNA looping formation [47], and in the amplification of distant estrogen response elements (DEREs) in some chromosomes [27], which seem to be important events in the altered expression of E2-related genes during tumorigenesis. Interestingly, a persistent stimulation with E2 can result in the loss of looping dynamics, inducing a permanent epigenetic silencing through hypermethylation of CGIs [26]. All of these studies provide information about the effect of the E2/ERα pathway in the activity of some chromatin-modifying enzymes (e.g., polycomb complex). Then, E2 can further recruit methylation machinery and trigger the DNA methylation process by regulating downstream genes in BC cells [28,48]. At the molecular level, those genes targeted and regulated by ERα must be identified, and, as the most challenging task, the architectures and underlying mechanisms of such regulation must be delineated. In summary, once the E2/ERα pathway is activated, it may induce the transcriptional expression or repression of E2-related genes. These results suggest an important role of the E2/ERα signaling pathway on genome organization and epigenetic silencing in several genes involved in breast tumorigenesis [49,50].

Our study provides the first evidence that *RPRM*, a potential p53-dependent tumor suppressor gene, is frequently hypermethylated in ERα-positive breast tumor types, probably due to the effect of E2/ERα in the recruitment of methylation components. Nevertheless, more studies are needed to examine this gain or loss of E2-dependent DNA methylation status in promoter CGIs, intragenic CGIs, intergenic CGIs, or non-CGIs promoters in order to understand the epigenetic tumoral biology and to identify potential markers to be used in clinics.

## 4. Materials and Methods

### 4.1. Tissue Samples

The methylation patterns in the 1.1 kb region (CGIs) of 77 primary tumors (≥70% tumor cellularity) and 10 normal breast tissue—obtained from normal individuals undergoing reduction mammoplasty—were analyzed in this study. Tumor tissues were collected from patients who underwent surgery in the Hospital Dr. Hernán Henríquez Aravena in Temuco, Chile, between 2002–2005, and stored at −80 °C for further processing. Clinicopathological data were collected from patient records and pathology reports. The mean age of this cohort was 58.14 years (standard deviation, 13.87 years). In addition, the mRNA expression levels of 18 primary tumors available from the same cohort used in MBDCap-seq (paired samples) were studied with the aim to correlate the mRNA expression with methylation data. From 18 primary tumors, 8 were classified as Luminal A, 6 of 18 as Luminal B, 3 of 18 as triple negative and 1 of 18 as HER2-positive. In order to validate the methylation results, we used a cohort of 26 BC samples: 15 BC ER-positive and 11 BC ER-negative. The mean age of this cohort was 56.26 years (standard deviation, 15.95 years).

### 4.2. DNA Methylation Profiles

The Cancer Methylome System (CMS) is a web-based database application designed for the visualization, comparison, and statistical analysis of human cancer-specific DNA methylation. This database was performed by the University of Texas Health Science Center at San Antonio (UTHSCSA) in collaboration with our research group. In fact, CMS was constructed using 77 breast tumors (donated by our research group), 10 normal breast tissues obtained through breast reduction, and 38 BC cell lines (provided by UTHSCSA). More details are exposed in the article of Gu et al. [29].

The DNA methylation intensities were directly quantified as the number of reads uniquely mapped to each 100-bp genomic bin. The comparative analysis of DNA methylation profiles among normal breast tissue, BC tissues, and cell lines was computationally performed. To do this, the datasets were downloaded from the CMS (http://cbbiweb.uthscsa.edu/KMethylomes/). Then, the mean methylation intensity of the CGIs region of *RPRM* gene (Start-End: chr2: 154042600–154043700 according to the UCSC Human Genome Browser “Human Mar. 2006 (NCBI36/hg18) Assembly”, length: 1.1 kb) was calculated in each case for the above-mentioned normal breast tissue, BC tissues, and cell lines. Finally, the methylation intensity of each group (normal breast tissue, BC, and cell lines) was compared using the appropriate statistical test.

### 4.3. Cell Line Culture

The BC cell lines used in this study were: T-47D, MDA-MB-231, BT-20, MCF7, and HCC1954, and the total RNA from the HMEC cells was generously provided by Tim Hui-Ming Huang (University of Texas Health Science Center at San Antonio, TX, USA). The MDA-MB-231 and BT-20 cells were cultured in High Glucose DMEM medium, the T-47D and HCC1954 cells were grown in RPMI-1640 medium, and the MCF7 cells were cultured in Advanced DMEM medium (Thermo Scientific, Waltham, MA, USA). All media were supplemented with 10% fetal bovine serum and 1% penicillin/streptomycin (Thermo Scientific, Waltham, MA, USA). The cell lines were incubated at 37 °C in a humidified atmosphere containing 5% CO_2_ and subculture during the logarithmic phase.

### 4.4. mRNA Expression by Real-Time PCR

Total RNA was isolated from cell lines and breast tissues using TRIzol reagent (Thermo Scientific, Waltham, MA, USA) according to the manufacturer’s instructions. First-strand cDNA was prepared from 1 μg of total RNA in a total reaction volume of 20 μL using M-MLV reverse transcriptase 200 U/µL (Promega, Madison, WI, USA) at 42 °C for 60 min. The qPCR analysis was performed using Brilliant II Ultra-Fast SYBR^®^ Green qPCR Master Mix according to the manufacturer’s protocol on the Stratagene Mx-3000p system (Agilent Technologies, Santa Clara, CA, USA). Relative expression was calculated by the 2^−ΔΔ*C*t^ methods, with *RNA18S5* and *ACTB* genes as controls. The primer sequences are detailed in Table 1.

### 4.5. Quantitative Methylation-Specific PCR (qMSP)

DNA extraction was performed using the Phenol-chloroform-isoamyl alcohol (25:24:1) method. Then, it was quantified in a nanodrop spectrophotometer to carry out the subsequent assays. Quantitative methylation-specific PCR, a qPCR-based method that measures fluorescent emission, was performed to determine the methylation levels of the promoter regions of *RPRM*. The primers and probe sequences used are shown in Table 2. The reaction was performed according to the following thermic profile: 95 °C for 10 min, followed by 40 cycles of 95 °C for 30 s, 56 °C for 1 min, and 72 °C for 30 s, using the Mx3000P QPCR System (Agilent Technologies, Santa Clara, CA, USA). Each PCR reaction included bisulfate-modified DNA samples, a 100% methylated DNA (Zymo Research, Irvine, CA, USA) as positive control, leukocyte DNA from a healthy person as negative control, and, finally, several blanks of PCR mix without DNA. Serial dilutions (250, 50, 10, 5, and 2 ng) of positive control were used for standard curve construction. The relative DNA methylation levels for *RPRM* were determined as the relation between the specific methylation of the amplified gene and *ACTB* (reference gene). The results were graphed using percentage-methylated relative (PMR; (methylation *RPRM*/methylation ACTB) × 100).

### 4.6. Ethics Statement

The Institutional Review Board of the School of Medicine of Universidad de La Frontera approved the collection, storage and use of samples for this study (Nº 20/011, December 2011).

### 4.7. Statistical Analysis

Data were analyzed by a Kruskal–Wallis test with Dunn’s post-test and the Spearman correlation test using the software SPSS v. 20 (SPSS Inc., Chicago, IL, USA). Also, we performed a Chi-square test or Fisher’s exact test and a Kaplan–Meier survival analysis. In order to properly perform these analyses, we defined the median value (50th percentile) of the methylation intensity of the CGIs promoter region as the cut-off point to group the cases as low methylation (LM) or high methylation (HM). These data were subsequently were converted to binary code 0 and 1, respectively. For the survival analysis, “survival” was defined as the time between the first surgery and the end of follow-up or death due to BC. Values were expressed as means +/− standard deviation (SD). Values of *p* < 0.05 were considered statistically significant.

## 5. Conclusions

Our data suggest that ERα expression in BC tissues is strongly associated with DNA methylation of CGIs in the *RPRM* gene. This is probably due to activation of the E2/ERα pathway and the subsequent activation/repression of ERα-dependent genes that modify the epigenetic state of DNA in tumor cells. This approach suggests that DNA methylation in the CGIs of certain tumor suppressor genes could be induced by E2 availability and a subsequent activation of the ERα pathway.

## Figures and Tables

**Table 1:** Association between *RPRM* methylation of CGIs and clinicopathological features.

Clinicopathological Features	*n*	Methylation of *RPRM* CGIs		*p*
		Low	High	
Age (year; mean 60)	77			0.021
≤60	43	23 (71.9%)	20 (44.4%)	
>60	34	9 (28.1%)	25 (55.6%)	
Tumor Size *	76			0.586
T1 + T2	58	23 (39.7%)	35 (60.3%)	
T3 + T4	18	9 (50.0%)	9 (50.0%)	
Lymph node metástasis *	76			0.247
No	35	12 (34.3%)	23 (65.7%)	
Yes	41	20 (48.4%)	21 (51.2%)	
TNM Stage *	76			0.621
I + II	51	20 (39.2%)	31 (60.8%)	
III + IV	25	12 (48.0%)	13 (52.0%)	
Elston Grade *	75			0.239
Well differentiated	14	5 (35.7%)	9 (64.3%)	
Moderately differentiated	34	12 (35.3%)	22 (64.7%)	
Poorly differentiated	27	15 (55.6%)	12 (44.4%)	
Estrogen receptor α *	74			0.000
ERα-negative	24	18 (75.0%)	6 (25.0%)	
ERα-positive	50	14 (28.0%)	36 (72.0%)	
Progesterone receptor *	74			0.034
PR-negative	35	20 (57.1%)	15 (42.9%)	
PR-positive	39	12 (30.8%)	27 (69.2%)	
Her2/*neu* *	65			1.000
Her2-negative	62	29 (46.8%)	33 (53.2%)	
Her2-positive	3	2 (66.7%)	1 (33.3%)	
Ki67 *	63			0.062
Low	50	19 (38.0%)	31 (62.0%)	
High	13	9 (69.2%)	4 (30.8%)	
Molecular Subtype *	67			0.001
Luminal A	30	6 (20.0%)	24 (80.0%)	
Luminal B	15	7 (46.7%)	8 (53.3%)	
Her2-positive	3	2 (66.7%)	1 (33.3%)	
TNBC	19	14 (77.8%)	4 (22.2%)	

* Several cases were excluded from that analysis due by missing information such as tumor size (1), lymph node metastasis (1), TNM stage (1), Elston grade (2), estrogen receptor α (3), progesterone receptor (3), Her2/*neu* (12), Ki-67 (14), and molecular subtype (10). *RPRM*, *Reprimo*.

**Table 2:** Primer and probe sequences used in this study.

ID	Sequences (5′-3′)	PCR Product (pb)	Ref.
*RPRM-M* (forward)	GCGAGTGAGCGTTTAGTTC	120	Sato et al. [13]
*RPRM-M* (reverse)	TACCTAAAACCGAATTCATCG	120	Sato et al. [13]
*B-actin-M* (forward)	TGGTGATGGAGGAGGTTTAGTAAGT	133	Moon et al. [51]
*B-actin-M* (reverse)	AACCAATAAAACCTACTCCTCCCTTAA	133	Moon et al. [51]
*RPRM* (probe qMSP)	/56-FAM/TT CGC GTC G/ZEN/T TCG CGG CGT TCG TT/3IABkFQ/	120	-
*β-actin* (probe qMSP)	/56-FAM/AC CAC CAC C/ZEN/C AAC ACA CAA TAA CAA ACA CA/3IABkFQ/	133	Moon et al. [51]

M = methylated form.

## Supplementary Materials

Supplementary materials can be found at www.mdpi.com/1422-0067/18/8/1525/s1.

## Abbreviations

RPRM	Reprimo
BC	Breast cancer
CGIs	CpG-islands
ERα	Estrogen receptor α
PR	Progesterone receptor
TNBC	Triple negative breast cancer
CMS	Cancer methylome system
MBD	Methyl-CpG binding domain
