# Supplementary material for: Reprimo, a Potential p53-Dependent Tumor Suppressor Gene, Is Frequently Hypermethylated in Estrogen Receptor α-Positive Breast Cancer
Source: Int J Mol Sci. 2017 Aug 15;18(8):1525. doi: 10.3390/ijms18081525 (PMC5577992; doi:10.3390/ijms18081525)
Supplement: Supplementary file 1 [file ijms-18-01525-s001.pdf]

Reprimo (RPRM), A Potential p53-Dependent Tumor Suppressor Gene is Frequently Hypermethylated in Estrogen Receptor α-Positive (ERα-Positive) Breast Cancer

Kurt Buchegger, Ismael Riquelme, Tamara Viscarra, Carmen Ili, Priscilla Brebi, Tim Hui-Ming Huang and Juan Carlos Roa

Table S1. Methylation intensity for each 100-bp of resolution in the RPRM CGIs of normal breast tissues, BC tissues and cell lines.

MBD-seq CGIs (chr2: 154042600 - 154043700)											
Phenotype	154042600	154042800	154042900	154043000	154043100	154043200	154043300	154043400	154043500	154043600	154043700
Normal Samples											
Normal-1	0	1	0	0	0	1	1	0	0	0	0
Normal-2	0	0	0	0	0	0	0	0	0	0	0
Normal-3	0	0	0	0	0	0	0	0	0	0	0
Normal-4	0	0	0	0	0	0	0	0	0	0	0
Normal-5	0	1	1	1	0	0	0	0	0	0	1
Normal-6	0	0	0	0	0	0	0	0	0	0	0
Normal-7	0	0	0	0	0	0	0	0	0	0	0

Normal-8	0	0	0	0	0	0	0	1	0	0	0
Normal-9	0	1	0	0	1	0	0	0	0	0	0
Normal-10	0	0	0	0	0	0	0	0	0	0	0
Breast Cancer Tissues											
Tumor-1	0	1	0	4	2	5	4	3	0	0	0
Tumor-2	5	7	2	3	2	2	3	4	0	0	1
Tumor-3	5	11	15	10	10	10	6	0	0	0	0
Tumor-4	33	60	53	35	31	27	23	11	0	0	0
Tumor-5	1	1	1	1	2	5	3	1	0	0	0
Tumor-6	2	5	8	0	6	2	0	0	0	1	0
Tumor-7	5	8	4	6	12	8	5	0	0	0	1
Tumor-8	2	4	3	13	15	19	7	6	2	1	0
Tumor-9	1	7	7	7	8	3	0	2	0	0	0
Tumor-10	5	23	32	11	15	18	13	12	9	7	6

Tumor-11	18	105	86	42	60	45	37	40	39	25	11
Tumor-12	0	1	0	1	0	0	0	0	0	0	0
Tumor-13	2	0	0	0	0	0	0	2	0	0	0
Tumor-14	2	1	2	3	0	1	1	0	0	0	0
Tumor-15	0	0	1	1	0	1	0	1	0	0	0
Tumor-16	4	11	5	14	3	2	5	4	5	7	2
Tumor-17	0	1	0	1	1	1	2	1	3	2	0
Tumor-18	1	3	3	2	6	2	2	2	0	0	1
Tumor-19	0	0	0	2	1	4	1	0	0	0	0
Tumor-20	0	0	1	0	0	0	2	0	0	0	0
Tumor-21	0	0	1	1	3	0	3	1	1	0	0
Tumor-22	0	0	0	0	0	0	0	0	0	0	0
Tumor-23	1	0	0	4	4	4	3	2	1	0	1
Tumor-24	0	8	0	10	3	3	0	2	0	0	0
Tumor-25	0	0	0	0	3	0	1	0	1	0	0

Tumor-26	0	0	1	1	4	5	6	1	0	1	0
Tumor-27	5	3	2	5	1	2	3	2	1	2	1
Tumor-28	1	0	0	0	1	1	0	0	0	0	0
Tumor-29	0	1	0	0	7	0	1	0	0	0	0
Tumor-30	5	22	21	7	11	7	8	2	2	1	1
Tumor-31	2	0	0	1	1	1	0	1	0	0	0
Tumor-32	1	1	1	1	2	0	1	1	1	0	0
Tumor-33	2	5	12	4	5	6	5	4	0	0	0
Tumor-34	10	5	5	5	3	0	0	1	0	0	0
Tumor-35	12	16	7	5	8	5	9	4	5	2	2
Tumor-36	2	0	3	2	0	0	0	1	0	0	0
Tumor-37	4	7	12	9	3	3	0	0	0	0	0
Tumor-38	0	1	0	1	1	2	1	0	0	0	1
Tumor-39	0	0	1	2	0	0	0	0	0	0	0
Tumor-40	0	0	0	5	1	3	2	0	0	0	0

Tumor-41	0	1	10	2	8	9	6	2	4	1	5
Tumor-42	4	10	14	5	23	13	17	11	6	7	4
Tumor-43	1	4	1	1	2	7	1	1	0	0	0
Tumor-44	0	1	0	0	0	0	1	0	0	1	0
Tumor-45	0	0	3	1	0	1	4	0	1	0	0
Tumor-46	1	7	10	16	18	5	14	7	4	4	6
Tumor-47	0	1	7	0	0	0	3	6	0	0	0
Tumor-48	5	11	11	4	0	0	0	0	0	0	0
Tumor-49	1	0	0	0	0	2	1	0	0	0	0
Tumor-50	1	0	2	1	0	4	2	0	0	0	0
Tumor-51	0	3	3	1	5	4	2	3	1	0	0
Tumor-52	1	0	2	0	0	1	0	0	0	0	0
Tumor-53	0	0	2	0	3	0	0	0	0	0	0
Tumor-54	0	0	2	0	0	0	0	0	0	0	0
Tumor-55	0	0	0	0	1	0	0	0	1	0	0

Tumor-56	1	11	7	9	5	3	3	11	10	7	4
Tumor-57	1	2	1	0	0	4	0	1	0	0	0
Tumor-58	1	1	5	5	7	6	2	3	0	0	0
Tumor-59	5	12	16	8	2	10	10	0	0	2	1
Tumor-60	0	0	1	0	1	0	0	0	0	0	0
Tumor-61	8	7	7	5	7	9	1	1	5	0	2
Tumor-62	0	1	2	0	0	1	4	0	0	0	0
Tumor-63	0	9	3	2	2	1	0	0	0	0	0
Tumor-64	2	0	0	0	0	1	0	0	0	0	0
Tumor-65	0	2	1	0	11	7	3	8	0	0	6
Tumor-66	4	20	25	7	3	6	7	4	3	1	3
Tumor-67	2	2	1	0	2	1	0	0	0	2	0
Tumor-68	0	3	4	0	1	2	0	1	0	0	0
Tumor-69	3	5	12	2	5	7	9	3	1	1	0
Tumor-70	0	2	1	0	5	5	2	1	0	0	0

Tumor-71	9	8	9	5	7	8	5	2	3	0	0
Tumor-72	1	1	2	0	0	2	0	3	0	0	0
Tumor-73	12	25	8	8	2	0	0	0	0	0	0
Tumor-74	0	0	0	0	0	0	0	0	0	0	0
Tumor-75	0	0	0	0	0	0	0	0	0	0	0
Tumor-76	1	0	1	1	1	2	1	2	1	0	2
Tumor-77	6	16	10	8	19	7	4	5	3	2	7
Breast Cancer Cell Lines											
184A1	0	7	5	6	1	3	1	0	0	0	1
AU565	1	5	4	1	0	0	0	0	0	0	0
BT549	1	12	4	1	1	1	3	1	0	0	0
HCC1395	1	0	3	0	2	4	1	0	0	0	0
HCC1569	7	1	0	2	4	10	8	4	0	0	0
HCC1937	1	1	1	4	4	2	0	1	0	0	0

HCC2185	0	0	3	1	0	1	0	0	0	0	0
HCC70	3	6	2	1	2	1	0	0	0	0	0
LY2	3	7	3	3	1	3	2	0	0	0	0
MCF-7	5	3	6	3	5	1	1	1	0	0	0
MDAMB-231	4	10	6	5	2	1	4	1	0	0	1
SUM149PT	2	0	4	0	2	2	3	0	1	0	0
SUM225CWN	4	1	7	2	0	0	0	0	0	0	0
T4	3	4	4	0	1	2	2	3	3	0	0
BT20	5	7	7	5	8	1	2	1	5	1	2
HCC1419	1	5	0	0	2	1	3	1	0	0	0
HCC1954	6	9	6	7	11	2	5	1	1	0	0
MCF10A	1	2	0	1	0	0	1	2	0	0	0
SKBR3	1	0	0	0	0	0	0	0	0	0	0
SUM159PT	2	4	4	1	2	2	1	1	0	0	0
BT474	0	1	1	0	0	0	0	0	0	0	0

HCC1143	0	0	0	0	0	0	0	0	0	0	0
HCC1428	2	0	2	0	2	0	1	0	0	0	0
HCC202	1	5	1	2	0	5	0	0	2	0	0
HCC3153	9	1	4	0	1	1	1	0	0	0	0
MDAMB436	3	7	2	0	1	2	0	0	0	0	0
SUM102PT	0	3	4	2	4	3	2	1	1	0	0
SUM185PE	0	2	0	1	1	1	3	0	1	0	0
600MPE	3	3	6	1	2	0	0	0	0	0	0
HCC1500	2	1	0	1	1	1	0	0	0	0	0
HCC1806	0	0	0	0	0	0	0	0	0	0	0
HS578T	1	6	3	1	1	2	0	0	0	0	0
MCF12A	6	8	19	9	8	4	2	4	4	2	0
MDAMB175VII	3	7	3	5	2	2	3	10	4	0	2
MDAMB453	7	8	11	10	2	3	4	3	5	1	1
SUM1315MO2	2	3	3	0	0	0	1	0	0	0	0

SUM52PE	11	11	7	3	2	7	2	2	6	2	0
MX-1	0	1	0	0	0	1	0	0	0	0	0

Table S2. Association between RPRM methylation of Promoter region and clinicopathological features.

**Percentage-methylated relative of CGIs RPRM**				
Clinicopathological features	*n*	Low	High	*p*
		32	45	
Age (year; mean 60)	26			0.134
≤ 60	19	12 (62.3%)	7 (36.8%)	
> 60	7	7 (100%)	0 (0.0%)	
Tumor Size *	25			0.724
T1	4	3 (75.0%)	1 (25.0%)	
T2	19	13 (68.4%)	6 (31.6%)	
T3	2	2 (100%)	0 (0.0%)	
Lymph node metastasis	26			1.000
No	16	12 (75.0%)	4 (25%)	
Yes	10	7 (70.0%)	3 (30.0%)	
Elston Grade	26			0.659
Well differentiated	2	2 (100%)	0 (0.0%)	
Moderately differentiated	11	8 (72.7%)	3 (27.3%)	
Poorly differentiated	13	9 (69.2%)	4 (30.8%)	
Estrogen receptor	26			0.010
ERα-negative	11	11 (100%)	0 (0.0%)	
ERα-positive	15	8 (53.3%)	7 (46.7%)	
Progesterone Receptor	26			0.665
PR-negative	14	11 (78.6%)	3 (21.4%)	
PR-positive	12	8 (66.7%)	4 (33.3%)	

* 1 case were excluded from that analysis due by missing information.

Table S3. Spearman correlation between methylation intensity of reads by each 100-bp and relative expression of RPRM mRNA.

	**MBDCap-seq CGIs (chr2: 154042600 - 154043700)**											
		154042600	154042800	154042900	154043000	154043100	154043200	154043300	154043400	154043500	154043600	154043700
Relative expression of RPRM (Log2)	*n*	18	18	18	18	18	18	18	18	18	18	18
	Correlation Coefficient	-0.479	-0.224	-0.348	-0.499	-0.390	-0.376	-0.475	-0.628	-0.544	-0.412	-0.444
	Significance	0.047	0.318	0.157	0.035	0.109	0.124	0.044	0.005	0.02	0.089	0.065
